# Effectiveness and cost-effectiveness of a minimal psychological intervention to reduce non-severe depression in chronically ill elderly patients: the design of a randomised controlled trial [ISRCTN92331982]

**DOI:** 10.1186/1471-2458-6-161

**Published:** 2006-06-21

**Authors:** F Lamers, CCM Jonkers, H Bosma, JPM Diederiks, JThM van Eijk

**Affiliations:** 1 Maastricht University, Faculty of Health Sciences, Department of Health Care Studies, section Medical Sociology, P.O. Box 616, 6200 MD Maastricht, The Netherlands

## Abstract

**Background:**

Depression is a prevalent disorder in chronically ill elderly persons. It may decrease quality of life, and increase functional disability, medical costs, and healthcare utilisation. Because patients may slip into a downward spiral, early recognition and treatment of depression is important. Depression can be treated with antidepressants or psychological interventions; the latter can also be applied by trained paraprofessionals.

In this paper, we describe the design of the DELTA study (Depression in Elderly with Long-Term Afflictions). The first objective of the DELTA study is to evaluate the effectiveness and cost-effectiveness of a minimal psychological intervention (MPI) to reduce depression in chronically ill elderly patients. The second objective is to evaluate whether a potential effect of the MPI may differ between types of chronic illnesses. The tailor-made intervention is administered by nurses, who are trained in the principles of cognitive behavioural therapy and self-management.

**Methods/Design:**

DELTA is a two-armed randomised controlled trial, comparing MPI to usual care. A total number of 180 patients with diabetes mellitus type II (DM) and 180 patients with chronic obstructive pulmonary disease (COPD), who in addition suffer from non-severe depression, will be included in the study. In our study, non-severe depression is defined as having minor depression, mild major depression or moderate major depression. The primary outcome measure is depression using the Beck Depression Inventory. Secondary outcome measures include quality of life, daily functioning, self-efficacy, autonomy, and participation. In the economic evaluation, cost-effectiveness and cost-utility ratios will be calculated. Furthermore, a process evaluation will be carried out.

Analyses will include both univariate and multivariate techniques and according to the intention to treat principle. The economic evaluation will be done from a societal perspective and data of the process evaluation will be analysed using descriptive techniques.

**Discussion:**

A total number of 361 patients has been included in the study. All interventions have been administered and follow-up data will be complete in September 2006.

Preliminary results from the process evaluation indicate that patients' satisfaction with the intervention is high. If this intervention proves to be effective, implementation of the DELTA intervention is considered and anticipated.

## Background

Depression is a prevalent and disabling disorder, especially in patients with chronic illnesses, such as diabetes mellitus type II (DM) and chronic obstructive pulmonary disease (COPD). In older patients with DM, prevalence rates of clinical relevant depression range from 14 to 17% [1,2]. In older patients with COPD, prevalence rates of 25% for minor depression have been reported [3]. Prevalence rates of major depression in older COPD patients range from 6 to 42% [4].

Persons suffering from minor or major depression have increased mortality risks and a decreased quality of life compared with non-depressed persons [5-7]. Furthermore, depression has been shown to increase health care utilisation [8,9], medical costs [10], and disability [8,9,11]. Since disability predicts the onset of depression and depression itself may further heighten risks of a progressing disability, this process of mutual reinforcement may lead to a downward spiral [11-14]. In addition, depression impairs one's ability to adhere to disease management regimens (diet, exercise, quitting smoking, taking medication regularly), potentially worsening the course of the chronic illness [15,16]. Hence, an early detection of depressive symptoms and treatment of depression is important in chronically ill elderly persons, thereby preventing or breaking a downward spiral. In primary care however, depression often remains undetected [17]. General practitioners have limited time and furthermore, current Dutch guidelines for DM and COPD don't take into account the psychological consequences of the chronic illness.

Available treatment options are antidepressants or psychological interventions. The effectiveness of antidepressants has been extensively studied and proven in major depression [18]. Since there is no clear evidence of the effectiveness of antidepressants in minor depression [19,20], clinical guidelines advise against using antidepressants in minor depression [21,22]. Cognitive therapy (CT) seems to be as effective as antidepressants in severe depression [23], and also in patients with mild and moderate depression [24]. Furthermore, CT seems to have an enduring effect [25]. It is also increasingly recognised that chronically ill elderly suffering from depression might benefit from psychosocial support and improving coping skills, such as self-management techniques [21,26]. In a study with DM type II patients, cognitive behavioural therapy (CBT) in combination with supportive diabetes education proved to be an effective treatment for major depression [27]. Similarly, self-management strategies in COPD patients have been reported to improve the patients' health status and to reduce hospital admissions [28].

Accumulating evidence shows that primary care staff can be trained in psychological interventions for depression [29]. Several studies reported that practice nurses can successfully administer interventions to reduce depression in primary care settings [30,31].

We developed a minimal psychological intervention (MPI), based on principles of self-management and CBT. The intervention is administered by nurses and aims to reduce non-severe depression in chronically ill elderly persons. Findings of a prior smaller pilot study showed that the intervention was feasible and acceptable to patients. Furthermore, the training programme, developed to teach nurses to administer the intervention, appeared to be feasible, attractive and successful among nurses [32].

In this contribution, we present the design of the DELTA study (Depression in Elderly with Long-Term Afflictions). The first objective of this randomised controlled trial (RCT) is to evaluate the effectiveness and cost-effectiveness of an MPI that is administered by a nurse and aims to reduce non-severe depression in chronically ill elderly patients. The effects of the MPI are compared with usual care. The second objective is to evaluate whether a potential effect of the MPI is different between types of chronic illnesses.

## Design and methods

### Design

The DELTA study is a two-armed randomised controlled trial, in which an effect evaluation, an economic evaluation and a process evaluation will be carried out. A total number of 360 patients will be included, 180 of which are patients with DM and 180 are patients suffering from COPD. We chose DM and COPD because first, they are highly prevalent in primary care. Second, they have a different course and prognosis. DM can be seen a gradual progressive illness, whereas COPD as a gradual relapsing condition [33]. This difference enables us to test whether the intervention is potentially generic. Approval for conducting this study was granted by the Medical Ethics Committee of the Maastricht University/Academic Hospital Maastricht.

### Setting and recruitment

In general practices in the southern part of Limburg, a province in the south of the Netherlands, all patients of 60 years and over with DM and or COPD were selected by the general practitioner, the general practitioner's assistant, or the research assistant. Selection was made using ICPC codes (T90, R91.01, R95, R99.06) if possible and otherwise by medication prescriptions (those drugs which are most often prescribed by the general practitioner for these chronic illnesses). In the last phase of patient selection, the general practitioner applied the inclusion and exclusion criteria using a pre-coded form with checkboxes for each criterion (Table 1).

All selected patients received a letter from their general practitioner with a request to complete a short screening questionnaire. This questionnaire, the Patient Health Questionnaire-9 (PHQ-9), consists of nine questions regarding the prevalence of symptoms of depression over the last two weeks. The response options are: "Not at all", "Several days", "More than half the days" and "Nearly every day". Its brevity and the fact that it is a self-administered questionnaire make it a useful tool in screening for depression in primary care. The PHQ-9 has been validated for both diagnosing depression and measuring severity [34-36]. Five questions on demographic variables were included in the questionnaire. Patients received a reminder by telephone two weeks after the questionnaire had been sent.

All patients who scored at least 2 depressive symptoms at least at "more than half the days" and at least one of these symptoms was depressed mood or anhedonia, were invited to participate in an interview to confirm or reject the diagnosis of depression.

The Mini International Neuropsychiatric Interview (MINI) was used to confirm the diagnosis from the PHQ-9. The interview took place at the patient's home and was administered by a trained nurse. The MINI is a validated and reliable diagnostic structured interview, covering 17 disorders based on DSM-IV criteria [37,38]. An extra diagnosis box for minor depression was added to the MINI, based on the research criteria for minor depression as described in the DSM-IV [39]. Furthermore, the Hamilton Depression Rating Scale (HDRS) was used to determine the severity of the depression [40,41]. Patients were excluded if they met one of the following criteria: if the MINI indicated a major depression in combination with a score above 18 (indicating a severe depression) on the HDRS, if the MINI indicated suicidal risk, or if the MINI indicated no depression at all (Table 1). Patients with a major depression and/or suicidal risk were referred back to their general practitioner. All remaining eligible patients (patients with a minor depression, non-severe major depression, or dysthymia) were invited to participate in the study and to give their informed consent.

### Randomisation

After having signed the informed consent form, patients enrolled in the study and filled in the baseline questionnaire. After having completed the baseline questionnaire, patients were assigned to either the intervention or control group. Randomisation was performed by an external agency using a computerized random number generator. In order to avoid an imbalance of chronic illness and general practice (care level) over the two groups, stratification for general practice and chronic illness (DM or COPD) was performed. Furthermore, to obtain equal numbers in both arms, a blocked design with a block size of two was applied. The intervention group received a Minimal Psychological Intervention, while the control group received usual care as given by their general practitioner, according to the guidelines for the specific chronic illness.

### Minimal Psychological Intervention

The intervention was given by a trained nurse, at the patient's own home. During a period of at most three months, patients received a maximum of 10 visits from the nurse. The number of visits depended on the patient's progress.

The Minimal Psychological Intervention contains elements from the Chronic Disease Self-Management Program (CDSMP) by Lorig and Gonzales [42], the Reattribution model from Goldberg [43] and from the work of the project group of the Interventie Studie Eerste Lijn (INSTEL) [44], as previously described [32]. The intervention aims at teaching patients to take responsibility for day-to-day management of their illness and its consequences. In short, it consists of five phases:

Phase 1. The nurse explores the patient's cognitions on the origin of symptoms and complaints, and their relation to limitations and behaviour.

Phase 2: The patient keeps a diary, where he or she records symptoms, complaints, thoughts, worries, related feelings, and behaviour.

Phase 3: Using information from the diary, the nurse challenges the patient to link his or her mood and consequent behaviour to the course of the chronic illness. A distinction will be made between complaints related to the illness itself, and those related to the emotional and behavioural consequences of the illness.

Phase 4: Introduction of the self-management approach by the nurse. The patient explores his or her possibilities to alter his or her behaviour. He or she then makes a plan on how to solve perceived problems and sets specific goals to be reached before the next visit from the nurse.

Phase 5: Evaluation of the progress in achieving the goals.

After a patient has completed these five phases successfully, he or she is supposed to be able to apply the self-management approach to any situation or problem he or she may encounter in the future. In consultation with the patient, the nurse can then decide to conclude the series of intervention visits.

### The training program for nurses

#### Administering the MINI

In an 8 h session, the nurses were trained how to confirm a diagnosis of depression by using the MINI and HDRS by a psychiatrist.

#### Applying the Minimal Psychological Intervention

During three 8 h sessions, with 2-week intervals, four nurses were trained by two experienced trainers (a psychologist/cognitive behaviour therapist, and a general practitioner) on how to apply the intervention. In between training days, nurses practised their newly learned skills on a pilot patient. As mentioned earlier, the training program has been shown to be feasible, attractive and successful among nurses [32]. Booster sessions were being held regularly during the study, and both a psychiatrist and a psychologist could be contacted by telephone to discuss cases at any time.

### Data collection

**Figure 1:**
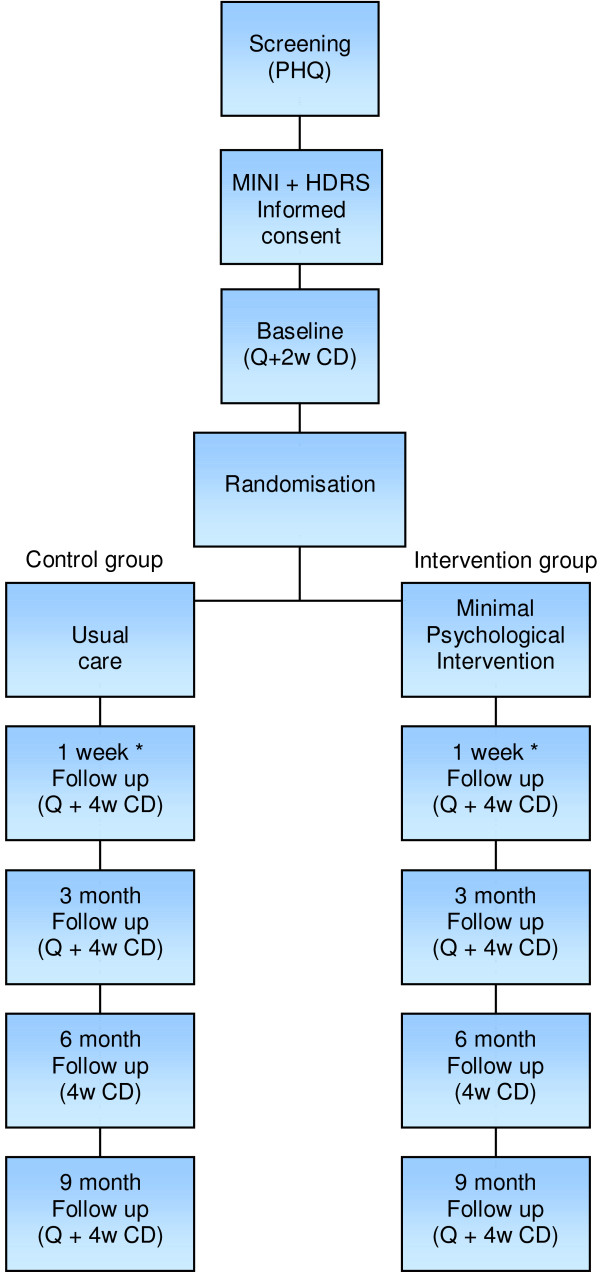
**DELTA flowchart**. Q = questionnaire, 2 w CD = two week cost diary, 4 w CD = four week cost diary * First follow up takes place one week after the intervention period. In the intervention group, this may vary from 2 weeks to three months. In the control group, the intervention period is fixed at six weeks, which is estimated to be the mean duration of the intervention in the intervention group.

Data was collected at five points in time: at baseline (T0), one week after the intervention period (T1), and at three, six and nine months after the intervention period (T2, T3, T4) (Fig. 1). The intervention period for patients allocated to the intervention group varies from one week to three months. The intervention period of the control group is fixed at six weeks, which is estimated to be the mean duration of the intervention period in the intervention group. Data were collected using self-administered questionnaires and cost diaries in combination with interviews by telephone.

### Effect evaluation

Table 2 provides an overview of the measures of the effect and economic evaluation, and time of assessment.

#### Primary outcome measures

The primary outcome measure in this study was level of depression, measured with the Beck Depression Inventory (BDI) [45,46]. The BDI consists of 21 items measuring symptoms of depression and has proven to be a valid and reliable tool [47].

#### Secondary outcome measures

Secondary outcome measures in the study were: Quality of life measured with the Short Form-36 (SF-36) [48], disease-specific quality of life assessed with the Problem Areas in Diabetes questionnaire (PAID-1) [49] for diabetes patients, and the St. George's Respiratory Questionnaire (SGRQ) for patients with pulmonary disease [50,51]. Furthermore, daily functioning was assessed with the Activities of Daily Life scale (ADL) from the Groningen Activity Restriction Scale (GARS) [52], self-efficacy assessed using the 12-item Self-efficacy scale [53,54] and autonomy and participation using the questions from the domain Autonomy outdoors from the Impact on Participation and Autonomy questionnaire (IPA) [55,56].

#### Covariates

Additionally, information on possible confounding factors and effect modifiers was collected. Information on demographic factors (age, gender, marital status, religion, education, occupation) was collected in the screening phase. Other factors measured are: coping using the active coping, avoidant coping and passive coping scales from the Utrecht Coping List (UCL) [57], mastery using the Personal Mastery Scale developed by Pearlin and Schooler [58], anxiety assessed using the anxiety subscale from the Symptom Checklist (SCL-90) [59], social support using the short version of the Social Support List- Interactions questionnaire (SSL-I) [60,61], co-morbidity using the Chronic conditions list from Statistics Netherlands (CBS – Centraal Bureau voor de Statistiek), life events using a list of 16 life events where patients report which life events they have experienced in the past year, and how they value these events (positive, negative, or neutral). Personality was measured using scales for neuroticism and extraversion from the Eysenck Personality Questionnaire (EPQ) [62]; severity of the chronic illness was assessed using the St. George's Respiratory Questionnaire (SGRQ) for COPD patients [50,51], and the Diabetes Symptom Checklist – Revised (DSC-R) for diabetes patients [63]. If possible, severity of the chronic illness will also be assessed by retrieving lung function (FEV_1_) and/or blood glucose levels (Hba1c) from hospital records or the general practitioner's records at the end of the study Finally, smoking and body mass index (BMI) were assessed, and in order to check for contamination in the control group, two questions to check whether or not the patients in the control group had heard or benefited from the intervention were added to the questionnaire. Contamination of the control group may lead to a smaller difference in effect between intervention and control group.

### Economic evaluation

A combined cost-effectiveness/cost-utility analyses will be performed from a societal perspective. The BDI is used as primary outcome measure in the cost-effectiveness analyses. The primary outcomes measure for the cost-utility measure will be utilities based on the social tariff of the EuroQol [64]. Healthcare costs, patient and family costs, as well as productivity losses will be recorded using cost diaries [65]. Patients prospectively kept the diary for two weeks at baseline and for four weeks at each follow up measurement. Afterwards, a telephonist contacted them to retrieve the information from the diary. Data were immediately entered in a computer file to ensure efficiency and reliability. The costs of the intervention were separately calculated. For the valuation of health care costs and patient and family costs, the updated Dutch Guideline for costing in economic evaluations [66] will be used. If no guideline costs existed, cost prizes were estimated using real costs and tariffs. For future costs and effectiveness data, a discount rate of 4% will be used.

### Process evaluation

A process evaluation was carried out to assess the following outcomes. The reach of the intervention, defined as the proportion of the intended target population that actually participated in the intervention. The dose delivered was defined as the completeness of the intervention and number and duration of the intervention visit. Dose received, described in two concepts, namely exposure and satisfaction. Exposure is the extent to which patients actively engage with and are receptive to the intervention, and satisfaction is defined as patient's satisfaction with the intervention [67]. Barriers were described as the extent in which problems were encountered during the intervention.

Data were collected using questionnaires filled out by nurses after every intervention visit, by means of checklists that were kept by the nurse for every patient to report which steps of the intervention had been taken, and by questionnaires filled out by patients after finishing the intervention.

### Analysis

Data will be analysed according to the intention to treat principle. In addition, on treatment analyses will be performed. Changes in primary and secondary outcome measures between intervention and control group will be analysed using both univariate and multivariate techniques. Models will be adjusted for age, gender and socio-economic status (SES), and baseline differences. Potential additional confounding factors and effect modifiers will be checked and, if necessary, included in the model. Since dependency between observations of subjects from the same general practice may exist as well as between repeated observations within persons, multilevel analyses will also be carried out.

All analyses will be performed for intervention and control group in total, as well as for DM and COPD separately.

In the economic evaluation the cost and effects of care as usual and MPI by a practice nurse will be calculated and compared. The cost-effectiveness ratio will be stated in terms of costs per improvement on the BDI, the cost-utility ratio will focus on the net cost per QALY gained. Ratios will be determined for the total patient population as well as for COPD or DM patients separately. Bootstrapping will be used to estimate confidence intervals for calculated ratios.

Descriptive statistics, Chi-square and t-tests will be used to analyse data from the process evaluation.

### Power calculation

Assuming an α of 0.05, a 1 – β (power) of 0.90, a decrease of 18 percent of non-severe depression in the intervention group versus zero percent in the control group, 192 persons were needed [68]; 48 COPD and 48 DM patients in the intervention group and 48 COPD and 48 DM patients in the control group. We decided to recruit four groups of 90 patients (in total: 360), as we not only anticipated the potential need for sub-group specific analyses, but also anticipated attrition varying between 20 and 30 percent (e.g. due to refusals during the follow-up).

## Discussion

### Progress of the study

Based on experiences in the pilot study, we anticipated having to screen 3600 patients in order to include 360 patients. However, we had to increase the number of patients to be screened to reach this number. This was done because the percentage of patients eligible for the MINI interview was lower than in the pilot study. Furthermore, the percentage of patients refusing the MINI interview was higher than expected. To arrive at a gross number of 360 patients we had to screen a total number of 8326 patients. The response rate to the screening questionnaire was 67%. Eventually, 361 non-severely depressed patients were recruited in the study (DM: N = 184; COPD: N = 177). All interventions have been administered; currently follow-up data are being collected. Data collection will be complete in September 2006.

### Process evaluation

First results of the process evaluation indicate that patients' satisfaction with the intervention is high, and 96.5% of the patients who received the intervention reported to have benefited from the intervention.

### Future implementation

If this intervention proves to be effective in reducing depression and improving quality of life and proves to be cost-effective, implementation of the intervention in the health care system is considered and anticipated. An implementation and dissemination plan has been developed and is regularly being updated to the latest insights.

## Competing interests

The author(s) declare that they have no competing interests.

## Authors' contributions

FL is investigator and wrote the manuscript, with input from the other authors. CJ is investigator, HB is supervising the planning and progress of the project, JD is co-author of the study protocol, and JvE is the principal investigator and author of the study protocol. All authors read, edited, and approved the final manuscript.

## Pre-publication history

The pre-publication history for this paper can be accessed here:



## Figures and Tables

**Table 1:** Inclusion and exclusion criteria as applied by the general practitioner* or research nurse^†^

**Inclusion criteria:**
Established diagnosis of Diabetes Mellitus Type II or COPD*
Age 60 years and over*
Community dwelling*
Minor depression or mild to moderate major depression according to MINI and HDRS criteria^†^
Completed informed consent^†^

**Exclusion criteria:**

Treatment with antidepressants*^†^
Severe major depression*^†^
Major psychiatric problems (bipolar depression, schizophrenia, suicidal risk)*^†^
Current psychosocial/psychiatric treatment*
Serious cognitive problems (demential syndrome)*
On waiting list for nursing home*
Bedridden*
Recent loss of spouse (< 3 months)*

**Table 2:** Outcome measures and time of assessment in the DELTA study

**Measure**	**Moment in time**						
*Name questionnaire/variable*	*Screening*	*Baseline*	*FU1*	*FU2*	*FU3*	*FU4*	*Other*

Marital status/living situation	x	-	-	-	-	-	-
Occupation/work situation	x	-	-	-	-	-	-
Education	x	-	-	-	-	-	-
Gender	x	-	-	-	-	-	-
Age	x	-	-	-	-	-	-
Religion	x	-	-	-	-	-	-

BDI	-	x	x	x	-	x	-
Euroqol (QALY's)	-	x	x	x	-	x	-
SF36	-	x	x	x	-	x	-
SGRQ	-	x	x	x	-	x	-
PAID-1	-	x	x	x	-	x	-
ADL-scale from GARS	-	x	x	x	-	x	-
SF36	-	x	x	x	-	x	-
UCL	-	x	x	x	-	x	-
Personal mastery scale	-	x	x	x	-	x	-
Self-efficacy-scale	-	x	x	x	-	x	-
IPA	-	x	x	x	-	x	-
SCL-90 subscale anxiety	-	x	x	x	-	x	-
SSL-I 12	-	x	-	-	-	x	-
CBS List Chronic conditions	-	x	-	-	-	x	-
Diabetes Symptom Checklist-Revised	-	x	x	x	-	x	-
Life-events	-	-	-	-	-	x	-
EPQ	-	-	x	-	-	-	-
Year of diagnosis DM/COPD	-	x	-	-	-	-	-
Smoking	-	-	-	-	-	x	-
BMI	-	-	-	-	-	x	-
Contamination in control group	-	-	-	-	-	x	-
Direct costs within health care system	-	x	x	x	x	x	-
Direct costs outside health care system	-	x	x	x	x	x	-
Indirect costs outside health care system	-	x	x	x	x	x	-

Lung function – if available	-	-	-	-	-	-	After intervention
Hba1c – if available	-	-	-	-	-	-	After intervention
Process evaluation	-	-	-	-	-	-	During nurses training program and intervention period
Compliance (in process-evaluation)	-	-	-	-	-	-	During intervention period

FU = follow up
